# Factors associated with fruit and vegetable consumption among Burmese refugees

**DOI:** 10.1017/S1368980023000125

**Published:** 2023-06

**Authors:** Hnin Wai Lwin Myo, Akiko S Hosler, Lawrence M Schell, Marie A Allsopp, Kaydian Reid

**Affiliations:** 1 Department of Epidemiology and Biostatistics, University at Albany, State University of New York, Health Sciences Campus, GEC 119, One University Place, Rensselaer, NY 12144, USA; 2 Department of Anthropology, University at Albany, State University of New York, Albany, NY, USA; 3 Department of Nutrition Science, Purdue University, West Lafayette, IN, USA; 4 Department of Nutrition and Public Health, University of Saint Joseph, West Hartford, CT, USA

**Keywords:** Fruits and vegetables, Burmese, Distance to food stores, Food shopping behaviour, Refugees

## Abstract

**Objective:**

The Burmese population is one of the fast-growing refugee populations in the USA. This study investigated behavioural and environmental factors associated with fruit and vegetable (FV) consumption among Burmese refugees.

**Design:**

We conducted a cross-sectional interview survey in 2018–2019. The 24-h recall was used to assess dietary behaviour. Multivariable logistic regression models were constructed with meeting the daily FV consumption recommendation (two or more servings of fruits and three or more servings of vegetables) as the outcome variable. We selected socio-economics, nutritional knowledge, food shopping frequency, ethnicity of preferred food store owners, perceived neighbourhood food environment and network distance to preferred food stores as potential explanatory variables.

**Setting:**

Two Upstate New York counties.

**Participants:**

Burmese refugees (*n* 173) aged ≥18 years.

**Results:**

Forty-five percentage of respondents met the daily FV consumption recommendation, and nearly all respondents identified ethnic (Burmese, Chinese/pan-Asian, or South Asian/halal) stores as their preferred stores to purchase FV. In the best-fit model, age (OR 1·08, 95 % CI (1·04, 1·12)) and shopping frequency (OR 1·51, 95 % CI (1·01, 2·26)) were positively associated, and network distance to preferred stores in kilometres (OR 0·81, 95 % CI (0·73, 0·90)) was negatively associated with meeting the daily FV consumption recommendation. No significant effect modifications by car ownership, poverty, length of stay in the USA and Supplemental Nutrition Assistance Program participation were detected.

**Conclusions:**

The findings suggested that having Asian ethnic food stores within a short, walkable distance from home and shopping at these stores often can promote healthy dietary behaviour among Burmese refugees.

In 2019, there were an estimated 189 000 Burmese individuals in the USA. This was more than a 10-fold increase since 2000 and the number is still growing, largely due to an influx of refugees who escaped the military dictatorship in Myanmar^(1)^. From 2008 to 2014 alone, approximately 117 000 Burmese refugees resettled in the USA, and 30 % went to states of Texas, New York or Indiana^(2)^. According to the 2017–2019 American Community Survey, Burmese individuals in the USA were mostly young adults aged between 18 and 49 years, with approximately two-thirds having an education of some high school or less and one-quarter in poverty^(1)^.

Upstate New York (NY) cities have growing Burmese resettlement communities. The Burmese consider food as an important source of health and well-being and believe some fresh fruits and vegetables have medicinal properties. Studies conducted in Buffalo and Albany, NY reported that most Burmese refugees perceived fresh fruits and vegetables were not accessible in their communities, and a lack of transportation to food stores was the most frequently mentioned barrier^(3,4)^. The limited availability of fresh produce in communities of low-income and/or racial minority individuals in Upstate NY has been widely reported^(4–8)^. Unfavourable neighbourhood food environment could be a major obstacle for Burmese refugees to maintain a healthy diet. However, research that examined the relationships between food environment measures such as distance to supermarkets and various dietary behaviours had mixed results and suggested complexity in the relationships and some methodological challenges^(9,10)^. There is a very limited body of literature on quantitative investigations of Burmese dietary behaviour.

To fill these knowledge gaps, this study examined factors associated with fruit and vegetable (FV) consumption among Burmese refugees in Upstate NY. Using the socio-ecological model of health as a conceptual framework, we hypothesised that healthy dietary behaviour would be determined by characteristics internal to the individual as well as layers of external or contextual influences. Specifically, we hypothesised that unfavourable neighbourhood food environment would be negatively associated with consumption of FV, while individual and interpersonal resources would be positively associated with consumption of FV. Methodologically, we focused on food resources that are culturally important and meaningful to Burmese refugees. The specific aim of this study was to build statistical models of the daily consumption of a recommended amount of FV among the Burmese refugees, with socio-economics, individual-level behaviour and knowledge (food shopping frequency and nutritional knowledge), interpersonal support (patronising Burmese-owned food stores) and food environment (perceived neighbourhood food environment, and distance to preferred food stores) as potential explanatory variables. We also investigated possible interactions of variables. The information gained from this study would contribute to understanding dietary behaviour of Burmese adults and also inform public health professionals about strategies to promote healthy dietary behaviour among Burmese refugees.

## Methods

We conducted a cross-sectional face-to-face interview survey in 2018–2019. Eligibility of the survey included a refugee/evacuee from Myanmar aged 18 years or older, a resident of Albany or Rensselaer Country in NY and being able to understand the consent written in English or Burmese. We sampled participants via purposive recruitment and referrals using the snowball method. Recruitment flyers were created with endorsement by local Burmese community leaders and distributed to community places where Burmese individuals gathered. A small group of known eligible individuals was also contacted via phone calls, e-mails and social networking services. We also recruited research volunteers including Burmese/English bilingual individuals from our university’s public health programmes. Through a power analysis (power = 85 % and *α* = 0·05), we learned that the minimum sample size needed would be 153. With an estimated attrition rate of 10 %, we determined that a sample size of 170 would be sufficient for this study. The questionnaire was written in English and Burmese. The accuracy of the translation was checked by the back-translation process. For a small number of Karen individuals with low English and Burmese proficiency, community leaders fluent in the Karen languages acted as interpreters. To have a convenient and comfortable interview environment, the survey was administered in a location each participant selected. There was no monetary incentive, but each survey participant received a MyPlate nutrition campaign promotional material.

The instruments and measures used in this study were as follows. FV consumption was measured by the 24-h dietary recall. We converted the reported FV intake into standard serving sizes based on the 2014 ASEAN Food Composition Database developed by Mahidol University in Thailand^(11)^. We selected this method because it provided the details of culturally relevant food intake patterns, while its respondent burden was relatively low^(11,12)^. A dichotomous FV consumption variable was created, with consuming two or more servings of fruits and three or more servings of vegetables per day as ‘yes’, and all other responses as ‘no’. This daily FV serving threshold is associated with lower all-cause and cause-specific mortality for serious chronic diseases^(13)^. It also closely approximates the daily FV consumption recommendation for adults by the US government’s MyPlate campaign^(14)^.

The socio-economic variables we selected for this study included age, sex, marital status, education, household income, length of stay in the USA, car ownership and participation in the Supplemental Nutrition Assistance Program (SNAP). Household income was converted to a dichotomous poverty variable. The 2019 US Federal Poverty Guidelines were used to determine the poverty status adjusted by family size.

Nutritional knowledge was assessed by the validated instrument used in a Chinese study by Wang *et al*.^(15)^ This instrument included 10 Likert-scale questions, and the summary score at or above the 50 percentiles (≥8) was considered to have satisfactory knowledge of general nutrition.

Food shopping behaviour was assessed by a series of questions about names, locations and shopping frequencies of places respondents usually shopped for FV. Respondents were also asked to identify one preferred store. We classified all reported stores into commonly recognised store types: supercentres, regional chain supermarkets, discount supermarkets, wholesale clubs (membership-based), ethnic stores and other. Ethnic stores were further grouped into Burmese, Chinese and South Asian (Indian, Pakistani or Bangladesh) based on the ethnicity of store owners.

Perceived neighbourhood food environment was assessed by three questions with ‘agree/disagreed’ responses. They were ‘Are the fruits and vegetables in your neighbourhood affordable?’ ‘Can you choose a variety of fruits and vegetables that you prefer in your neighbourhood?’ and ‘Are these fruits and vegetables of good quality?’ The perceived neighbourhood food environment variable was coded ‘favourable’ if the respondents agreed to all three questions. As validation against an objectively measured food environment, we assessed agreement between the unfavourably perceived neighbourhood food environment and the 2019 low-income/low-food access census tract designation by the US Department of Agriculture (USDA)^(16)^. Finally, we obtained the network walking distances between respondents’ homes and all food shopping venues using online Geographic Information System resources.

We constructed multivariable logistic regression models for the daily consumption of two or more servings of fruits and three or more servings of vegetables and used the stepwise backward deletion method to determine the best-fit model. The Hosmer–Lemeshow test was used to evaluate the model fit, with a *P*-value of <0·05. We also evaluated models with and without several combinations of interactions using the Wald *χ* ^2^ test and the likelihood ratio test. We reported the OR, 95 %CI and the *P*-value of each variable that remained in the models. SPSS (version 28.0; IBM, Corp., 2021) was used for statistical analysis.

## Results

We contacted 189 potentially eligible Burmese individuals, and 182 agreed to participate in the study. Three individuals moved outside the state before the interviews, and six individuals did not complete the interviews. A total of 173 participants were included in the sample. Table 1 presents the socio-economic characteristics of the sample. Briefly, a majority of respondents (54·3 %) were 35–54 years of age. The sex ratio was balanced with 52·0 % female, and three-quarters were married or living with a partner. Nearly 70 % had the highest education of some high school or less, and 27·7 % were in poverty. About two-thirds were in the USA for less than 10 years, 54·3 % did not own a car and 34·7 % received SNAP benefits.

**Table 1:** Socio-economic characteristic of the upstate NY Burmese refugee sample (*n* 173)

Characteristic		*n*	%
Age	18–34	37	21·4
	35–54	94	54·3
	55+	42	24·3
Sex	Female	90	52·0
	Male	83	48·0
Marital status	Married or with a partner	131	75·7
	Not married or without a partner	42	24·3
Education	Some high school or less	121	69·9
	High school graduate or higher	52	30·1
Poverty (adjusted by household size)	In poverty	48	27·7
	Not in poverty	125	72·3
Length of stay in the USA	Less than 10 years	117	67·6
	10 years or longer	56	32·4
Car ownership	Yes	79	45·7
	No	94	54·3
SNAP participation	Yes	60	34·7
	No	113	65·3

SNAP, Supplemental Nutrition Assistance Program.

Approximately 45 % of respondents met the FV recommendation of consuming two or more servings of fruits and three or more servings of vegetables a day, and a similar proportion of respondents (43·9 %) had satisfactory general nutritional knowledge (Table 2). However, a large majority (82·1 %) perceived their neighbourhood food environment as unfavourable. In addition, 86·1 % of respondents resided in the census tracts identified as low-income and low-food access. The perceived and the objectively measured neighbourhood food environments had a good agreement, with Gwet’s AC1 (a chance-corrected measure of agreement) of 0·79 (95 % CI (0·71, 0·88)).

**Table 2:** Nutrition and food shopping behaviour of the upstate NY Burmese refugee sample (*n* 173)

Characteristic		*n*	%
Meeting the daily fruit and vegetable consumption recommendation^*^	Yes	78	45·1
	No	95	54·9
Nutritional knowledge	Satisfactory	76	43·9
	Not satisfactory	97	56·1
Perceived neighbourhood food environment	Favourable	31	17·9
	Not favourable	142	82·1
Preferred food store	Burmese	91	52·6
	Chinese (pan-Asian)	65	37·6
	South Asian (halal)	16	9·2
	Regional supermarket	1	0·6
Shopping frequency at preferred store	Once a month	6	3·5
	2–3 times a month	34	19·7
	Once a week	60	34·7
	2 or more times a week	73	42·2
Network distance to preferred store	Less than 1·3 km (0·8 mile)	54	31·2
	1·3 km (0·8 mile) or longer	119	68·8

* Consuming two or more servings of fruits and three or more servings of vegetables per day.

In terms of food shopping behaviour, a total of twenty-five stores were named and included four supercentres, eight regional supermarkets, three discount supermarkets, two wholesale clubs and eight ethnic stores. The mean network distance from home and its sd for each type of food shopping venue were as follows: supercentres 6·7 km (sd 2·8 km), regional supermarkets 4·9 km (sd 2·7 km), discount supermarkets 2·2 km (sd 2·0 km), wholesale clubs 11·6 km (sd 3·0 km) and ethnic stores 4·3 km (sd 2·6 km). All respondents identified at least one ethnic store as their usual place to shop for FV. Furthermore, nearly all respondents (99·4 %) identified an ethnic store as their preferred food store. Stores owned by Burmese individuals selling Burmese food were the most frequently identified preferred stores (52·6 %), followed by Chinese-owned stores selling pan-Asian foods (37·6 %) and South Asian-owned stores selling halal products (9·2 %). In terms of shopping frequency at preferred stores, 42·2 % reported 2 or more times a week, and 34·7 % reported once a week. Finally, network distances to preferred stores ranged from less than 0·1 km to 17·6 km, with the mean of 3·6 km (sd 2·2 km). Close to a third of respondents (31·2 %) lived less than 1·3 km (0·8 mile) from their preferred food stores or approximately less than 15 min of travel time by walking.

Table 3 presents the results of multivariable logistic regression analyses. Model 1 is the intermediate model that contained at least one variable from each of the four domains (socio-economic, behaviour and knowledge, interpersonal and environment) in the stepwise backward deletion process. Model 2 is the best-fit model, and it indicates that meeting the daily FV consumption recommendation was positively associated with age (OR 1·08, 95 % CI (1·04, 1·12)) and shopping frequency at preferred stores (OR 1·51, 95 % CI (1·01, 2·26)) and negatively associated with network distance to preferred stores in kilometres (OR 0·81, 95 % CI (0·73, 0·90)). Marital status and education remained in the best-fit model, but they were not statistically significant (*P* > 0·05). Furthermore, no significant effect modifications by car ownership, poverty, length of stay in the USA and SNAP participation were detected.

**Table 3:** Multivariable logistic regression models for meeting the daily fruit and vegetable consumption recommendation*: a sample of Burmese refugees (*n* 173)

Variable	Model 1 (intermediate)				Model 2 (best-fit)			
	OR	95 % CI		*P*	OR	95 % CI		*P*
		Lower	Upper			Lower	Upper	
Socio-economics								
Age	1·08	1·05	1·12	<0·001	1·08	1·04	1·12	<0·001
Sex (Male)	0·74	0·36	1·54	0·428				
Marital status (not married/without partner)	0·36	0·13	0·99	0·049	0·43	0·17	1·10	0·079
Years of education	1·05	0·97	1·14	0·249	1·07	0·99	1·16	0·093
Behaviour and knowledge								
Shopping frequency	1·43	0·94	2·17	0·099	1·51	1·01	2·26	0·043
Nutritional knowledge	1·13	0·86	1·50	0·389				
Interpersonal								
Ethnicity of preferred store owner								
South Asian and other (Ref)								
Chinese	1·15	0·42	3·14	0·799				
Burmese	1·33	0·37	4·81	0·663				
Environment								
Perceived NFE	1·11	0·94	1·31	0·219				
Network distance to preferred store	0·80	0·70	0·92	0·001	0·81	0·73	0·90	<0·001

NFE, neighbourhood food environment.

* Consuming two or more servings of fruits and three or more servings of vegetables per day.

## Discussion

This study was one of the very few quantitative investigations of the dietary behaviour of Burmese refugees in the USA. Several new and unique findings have been presented by this study. First, the proportion of Burmese adults meeting the daily FV consumption recommendation (45·1 %) was considerably higher compared to the general population. For instance, 12·2 and 9·3 % of US adults met the FV consumption recommendations, respectively, in 2015, based on the 6-item food frequency questions by the Behavioural Risk Factor Surveillance System surveys^(17)^. A study that used the averaged 2-d dietary recall by the 2007–2012 National Health and Nutrition Examination Survey reported that 21·6 and 28·7 % of US adults met the FV recommendations, respectively^(18)^. Nonetheless, significant disparities in FV consumption existed among the Burmese respondents.

The present study revealed that age was one of the independent factors associated with FV consumption. Positive associations between age and FV consumption in US adult populations have been reported^(17,19)^. Literature suggests that increased consumption of FV at older age is associated with a diagnosis of serious chronic disease such as cancer or diabetes, which strengthens commitment to eat healthfully^(19)^. It is also argued that older adults differ from their younger counterparts in the degree to which fruit and vegetables are considered priority purchases^(20)^. For instance, food scarcity and agrarian life experience in childhood may be associated with older adults’ tendency to purchase and consume more fruits and vegetables than younger adults. Among Burmese refugees, older adults tend to adhere to traditional home-prepared Burmese food, which uses generous amounts of fresh vegetables, herbs and tropical fruits.

Almost all Burmese participants in this study reported Asian ethnic stores as their preferred stores to shop for FV. A strong preference for ethnic stores by new immigrants has been reported by studies conducted in Upstate NY^(4,21)^. Supermarkets are known for their dominance in the retail fresh produce business through strategic pricing^(22)^, and they have been the primary focus of food environment research in the USA^(16)^. On the other hand, ethnic stores are perceived as an insignificant source of healthy foods^(23)^. However, ethnic stores are more resilient than supermarkets. Ethnic stores survive in low-income communities where supermarkets have fled, because they can take advantage of low rents and low wages of family and co-ethnic workers and stay competitive by having a channel of suppliers who have exclusive access to ethnic grocery^(24)^. For immigrants who are at the low level of dietary assimilation and reside in neighbourhoods with unfavourable food environments, ethnic stores are increasingly important sources of FV^(8,23)^. A longitudinal study of the food environment in Albany, NY reported that ethnic stores were generally ‘healthier’ stores compared to convenience stores and dollar/discount stores, because they stocked a greater variety of fresh produce and did not sell alcohol and tobacco^(8)^. A study conducted in nearby Schenectady, NY revealed that shopping at ethnic stores was associated with lower BMI among low-income Indo-Guyanese immigrants^(21)^. Many respondents in this study mentioned that Asian ethnic stores were the only places where they could find a variety of tropical fruits, fresh greens, herbs and spices they frequently use in home cooking. Some of them would walk more than a half hour to get to their favourite ethnic stores, even though supermarkets were located closer to their homes. Respondents also mentioned a familiar, welcoming atmosphere of immigrant-owned stores as an important reason for patronising ethnic stores.

Interestingly, the ethnicity of store owners did not have a significant association with FV consumption. The finding indicated that interpersonal interaction with a Burmese store owner, who shares the same language, cultural background and refugee experience, was not a determinant for greater FV consumption. It may be related to the fact that Burmese-owned stores tended to be small family-owned stores, and although they stocked hard-to-get Burmese fresh produce, they often lacked consistency in quality and quantity. Some Burmese respondents therefore preferred larger, well-stocked Chinese-owned pan-Asian food stores or South Asian-owned stores where selections of herbs and spices were greater.

The present study demonstrated that greater shopping frequency and shorter distance to preferred stores were independently associated with meeting the daily FV consumption recommendation. As for shopping frequency, a systematic review of twenty-four research papers supported the positive association between the frequency of food shopping and FV consumption^(25)^. The role of the distance to stores however remains unsettled. Existing literature has confirmed that preferred or primary food stores are not the nearest food stores for most Americans, and the measured distance to the preferred stores has no consistent association with FV consumption^(9,10,26,27)^. For instance, a Seattle study reported FV consumption was not associated with the distance to the primary food stores but with the cost range of merchandise^(26)^. A New Orleans study concluded that FV consumption was not associated with the distance to the primary store or car access but with the number of monthly shopping trips^(27)^. It should be noted that respondents of these previous studies overwhelmingly preferred supermarkets, and their car ownership was about twice higher (85·5–92·3 %) compared to Burmese respondents in the present study (45·7 %). The average distance to preferred stores for Burmese respondents (3·6 km) was considerably shorter compared to 6·1 km for the average American^(28)^. Furthermore, a great majority of Burmese respondents perceived their neighbourhood food environment as unfavourable, and this perception was supported by the objectively measured low-income/low-food access census tract designations by the USDA. The unique spatial and socio-economic environments Burmese refugees lived in need to be considered when interpreting the findings.

Limitations exist in this study. Convenience sampling used in this study can introduce selection bias. We acknowledge, however, that the age, sex, education and poverty distributions of Burmese respondents in this study were very similar to those of the Burmese population in the USA^(1)^, indicating good overall representativeness and generalisability. The 24-h recall method to measure FV consumption has intrinsic limitations, including under-reporting of energy due to social desirability, and day-to-day variation, which is a within-person random error. A lack of validation studies specific to the Burmese population is a limitation of this study^(29)^. The 24-h recall, however, is appropriate for use with individuals with low literacy and distinctive food cultures^(12)^. Finally, as a cross-sectional study, the directions of associations cannot be established.

### Implications for research and practice

The findings of this study strongly suggest that Asian ethnic food stores, regardless of the ethnicity of store owners, are important sources of FV for Burmese refugees. The findings also suggest that having ethnic stores within a short, walkable distance and shopping at these stores often can promote FV consumption. Partnering with the existing ethnic stores in the study community appears to be the most appropriate and sustainable approach to increase FV consumption among Burmese refugees.

Public health intervention ideas include strategies to promote ethnic food businesses, for instance, assisting them in purchasing better refrigeration equipment, improving in-store display and advertisement and setting up online shopping options. At the community level, providing frequent transportation between Burmese residential areas and ethnic stores and improving pedestrian safety near the stores can improve spatial access. Because all the existing ethnic stores in the study community are authorised SNAP vendors, assisting eligible Burmese families to enrol in SNAP is also important. For any approach, minimising communication and cultural barriers is essential.
